# PRDX2 and PRDX4 are negative regulators of hypoxia-inducible factors under conditions of prolonged hypoxia

**DOI:** 10.18632/oncotarget.7142

**Published:** 2016-02-02

**Authors:** Weibo Luo, Ivan Chen, Yan Chen, Duah Alkam, Yingfei Wang, Gregg L. Semenza

**Affiliations:** ^1^ Vascular Program, Institute for Cell Engineering, Johns Hopkins University School of Medicine, Baltimore, MD, USA; ^2^ Department of Biological Chemistry, Johns Hopkins University School of Medicine, Baltimore, MD, USA; ^3^ Department of Oncology, Johns Hopkins University School of Medicine, Baltimore, MD, USA; ^4^ Department of Pediatrics, Johns Hopkins University School of Medicine, Baltimore, MD, USA; ^5^ Department of Medicine, Johns Hopkins University School of Medicine, Baltimore, MD, USA; ^6^ Department of Radiation Oncology, Johns Hopkins University School of Medicine, Baltimore, MD, USA; ^7^ Department of McKusick-Nathans Institute of Genetic Medicine, Johns Hopkins University School of Medicine, Baltimore, MD, USA; ^8^ Department of Pathology, UT Southwestern Medical Center, Dallas, TX, USA; ^9^ Department of Pharmacology, UT Southwestern Medical Center, Dallas, TX, USA; ^10^ Department of Neurology and Neurotherapeutics, UT Southwestern Medical Center, Dallas, TX, USA

**Keywords:** PRDX2, PRDX4, HIFs, transcriptional corepressor

## Abstract

Hypoxia-inducible factors (HIFs) control the transcription of genes that are crucial for the pathogenesis of cancer and other human diseases. The transcriptional activity of HIFs is rapidly increased upon exposure to hypoxia, but expression of some HIF target genes decreases during prolonged hypoxia. However, the underlying mechanism for feedback inhibition is not completely understood. Here, we report that peroxiredoxin 2 (PRDX2) and PRDX4 interact with HIF-1α and HIF-2α *in vitro* and in hypoxic HeLa cells. Prolonged hypoxia increases the nuclear translocation of PRDX2 and PRDX4. As a result, PRDX2 and PRDX4 impair HIF-1 and HIF-2 binding to the hypoxia response elements of a subset of HIF target genes, thereby inhibiting gene transcription in cells exposed to prolonged hypoxia. PRDX2 and PRDX4 have no effect on the recruitment of p300 and RNA polymerase II to HIF target genes and the enzymatic activity of PRDX2 and PRDX4 is not required for inhibition of HIF-1 and HIF-2. We also demonstrate that PRDX2 is a direct HIF target gene and that PRDX2 expression is induced by prolonged hypoxia. These findings uncover a novel feedback mechanism for inhibition of HIF transcriptional activity under conditions of prolonged hypoxia.

## INTRODUCTION

Hypoxia-inducible factors (HIFs) are master regulators of transcriptional responses to reduced O_2_ availability [1]. HIFs are heterodimeric transcription factors consisting of α and β subunits [2]. Three α subunits (HIF-1α, HIF-2α, and HIF-3α) and one β subunit (HIF-1β) have been identified [2-7]. HIF heterodimers bind to the consensus nucleotide sequence 5′-(A/G)CGTG-3′ [8] found within hypoxia response elements (HREs) and modulate the transcription of over 1500 genes, whose protein products regulate a wide range of biological processes, including angiogenesis, energy metabolism, pH homeostasis, cell survival and proliferation, DNA repair, immune evasion, tumor invasion and metastasis [9, 10]. HIFs represent targets for therapy in many human diseases, including anemia, cancer, cardiovascular diseases, diabetes, ocular diseases, organ transplant rejection, polycythemia, pulmonary hypertension, sleep apnea, ulcerative colitis, and wound healing [9]. Understanding the regulatory mechanisms underlying HIF transcriptional activity may yield novel therapeutic targets.

Previous studies have demonstrated the complexity of negative regulation of HIF activity. In well-oxygenated cells, HIF-α subunits are hydroxylated on two proline residues (P402 and P564 of human HIF-1α; P405 and P531 of human HIF-2α). Proline hydroxylation is required for binding of the von Hippel-Lindau protein (VHL), which recruits the Elongin B/C E3-ubiquitin protein ligase complex that mediates ubiquitination and subsequent degradation of HIF-α subunits in the 26*S* proteasome [11-14]. OS-9 is a protein that interacts with both HIF-1α and PHD2 to promote proline hydroxylation [15], whereas SSAT2 interacts with HIF-1α, VHL, and Elongin C to promote hydroxylation-dependent ubiquitination [16]. MCM7 also interacts with HIF-1α, VHL, and Elongin C to enhance ubiquitination and degradation of HIF-1α [17].

HIF-1α protein stability is also regulated by oxygen-independent mechanisms. The ubiquitin E3 ligase CHIP cooperates with HSP70 to induce HIF-1α protein degradation in the 26*S* proteasome during prolonged hypoxia [18]. HAF is another ubiquitin E3 ligase that mediates proteasome-dependent HIF-1α protein degradation and decreases HIF-1 activity [19]. BHLHE41 (also known as SHARP1) binds to, and promotes VHL-independent proteasomal degradation of HIF-1α and HIF-2α [20]. HSP90 inhibitors increase the ubiquitination and proteasomal degradation of HIF-1α that is triggered by binding of RACK1 at the site vacated by HSP90 [21]. SSAT1 binds to both HIF-1α and RACK1 to promote ubiquitination of HIF-1α [22]. The tumor suppressor p53 also binds to HIF-1α and induces MDM2-dependent ubiquitination and proteasomal degradation of HIF-1α [23]. Finally, HIF-1α is also subject to lysosomal degradation through chaperone-mediated autophagy, which is mediated by binding of HSC70 and LAMP2A [24].

In addition to the regulation of protein stability, the transcriptional activity of HIF-1α is O_2_-regulated by factor inhibiting HIF-1 (FIH-1) [25], which catalyzes asparagine hydroxylation (N803 of human HIF-1α; N847 of human HIF-2α) that inhibits interaction of HIF-1α with the coactivator p300, thereby blocking a step that is necessary for transactivation [25-27]. MCM3 interacts with HIF-1α (and HIF-2α) and inhibits transactivation in an asparagine hydroxylation-dependent manner [17]. EAF2 disrupts p300 recruitment to suppress HIF-1 transactivation, which is independent of FIH-1 [28]. Four-and-a-half LIM domain protein 2 (FHL2) interacts with the HIF-1α transactivation domain to repress its transcriptional activity [29]. Reptin interacts with HIF-1α to inhibit transactivation of a subset of HIF target genes [30]. Sirt1 deacetylates HIF-1α at lysine 674 to block p300 recruitment and subsequent HIF-1 target gene transcription [31], whereas deacetylation of HIF-2α by Sirt 1 augments HIF-2 transcriptional activity [32]. However, Sirt1 was also reported to increase HIF-1α protein stability [33]. Sirt6 functions as a co-repressor of HIF-1 to regulate glucose homeostasis in mice [34]. Sirt7 is also a negative regulator of HIF-1 and HIF-2 [35]. Thus, a complex array of protein-protein interactions controls HIF stability and transcriptional activity.

The peroxiredoxin (PRDX) family of peroxidases is abundantly expressed in cells and metabolizes intracellular H_2_O_2_ through the thioredoxin system [36]. In mammals, there are six family members (PRDX1-6), which are divided into three subgroups according to their catalytic mechanism: typical 2-cysteine PRDX (PRDX1-4), atypical 2-cysteine PRDX (PRDX5), and 1-cysteine PRDX (PRDX6) [36]. Hypoxia induced PRDX1 expression in oral squamous carcinoma SCC15 cells [37], whereas HIF-1 suppressed PRDX3 expression in VHL-deficient clear cell renal carcinoma cells [38]. PRDX1 functioned as a ligand for Toll-like receptor 4 to enhance HIF-1α expression and HIF-1 binding to the promoter of the *VEGF* gene in endothelial cells, thereby potentiating VEGF expression [39]. Expression of PRDX5 targeted to the mitochondrial intermembrane space decreased hypoxia-induced reactive oxygen species and attenuated HIF-1α protein levels and HIF-1 target gene expression in rat pulmonary artery smooth muscle cells [40]. Regulation of HIF activity by PRDX2 or PRDX4 has not been reported.

In the present study, we demonstrate that several PRDX family members directly interact with HIF-1α and HIF-2α in hypoxic human HeLa cells. PRDX2 and PRDX4 suppress transcription of a subset of HIF-1 and HIF-2 target genes under conditions of prolonged hypoxia. *PRDX2* is a novel HIF target gene and hypoxia-induced PRDX2 expression results in feedback inhibition of HIF activity in HeLa cells subjected to prolonged hypoxia.

## RESULTS

### Identification of PRDX family members as novel HIF-1α- and HIF-2α-interacting proteins

**Figure 1:**
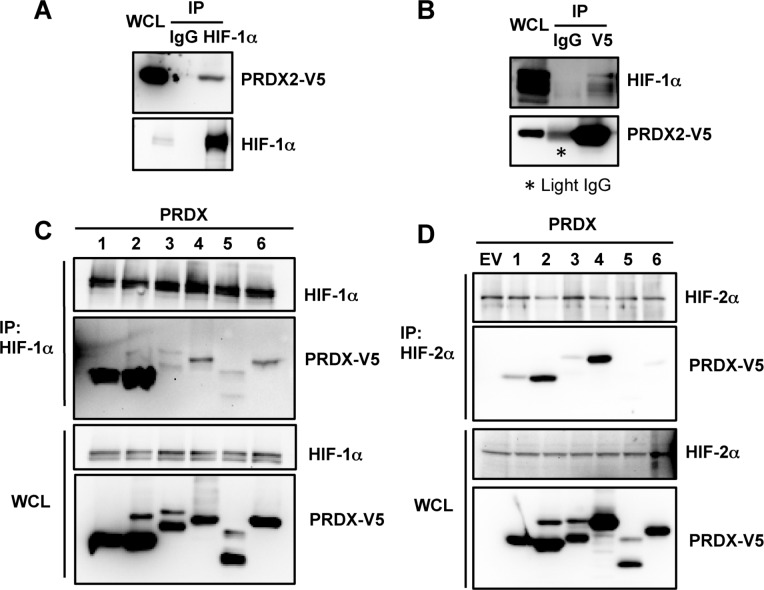
PRDX proteins bind to HIF-1α and HIF-2α **A.** HeLa cells were transfected with an expression vector encoding V5-epitope-tagged PRDX2 (PRDX2-V5) and exposed to 1% O_2_ for 24 h. Whole cell lysate (WCL) was subject to immunoprecipitation (IP) using anti-HIF-1α antibody or control IgG, followed by immunoblot assays with antibody against V5 epitope or HIF-1α. **B.** HeLa cells were transfected with PRDX2-V5 vector and exposed to 1% O_2_ for 24 h. The WCL was subject to IP using anti-V5 antibody or control IgG, followed by immunoblot assays with antibody against V5 or HIF-1α. Light IgG: immunoglobulin light chain from the secondary antibody. **C.** HeLa cells were transfected with vector encoding a V5-tagged PRDX family member and exposed to 1% O_2_ for 24 h. WCL was subject to IP using anti-HIF-1α antibody, followed by immunoblot assays with antibody against V5 or HIF-1α. **D.** HeLa cells were transfected with empty vector (EV) or vector encoding a V5-tagged PRDX family member and exposed to 1% O_2_ for 24 h. WCL was subject to IP using anti-HIF-2α antibody, followed by immunoblot assays with antibody against V5 or HIF-2α.

Our previous proteomic screening identified several positive and negative regulators that directly control the activity of HIF-1 and HIF-2 [17, 41, 42]. PRDX2 was also identified as a candidate HIF-1α-interacting protein in the screen. To validate the screening data, HeLa cells were transfected with an expression vector encoding V5-epitope-tagged PRDX2 and exposed to 1% O_2_ for 24 h. Anti-HIF-1α antibody co-immunoprecipitated PRDX2-V5 protein from hypoxic HeLa cell lysates (Figure 1A). Conversely, endogenous HIF-1α was co-immunoprecipitated from hypoxic cell lysates by anti-V5 antibody (Figure 1B). These data indicate that PRDX2 interacts with HIF-1α in HeLa cells.

To determine whether other PRDX family members also bind to HIF-1α, we performed immunoprecipitation (IP) assays using whole cell lysates (WCLs) prepared from transfected HeLa cells that were exposed to hypoxia. As shown in Figure 1C, PRDX1-V5 strongly interacted with endogenous HIF-1α, similar to PRDX2-V5, whereas PRDX4-V5 and PRDX6-V5 weakly interacted with endogenous HIF-1α. In contrast, PRDX3-V5 and PRDX5-V5 failed to bind to HIF-1α. These data indicate that PRDX1, PRDX2, PRDX4, and PRDX6, but not PRDX3 or PRDX5, interact with HIF-1α when overexpressed in HeLa cells.

Next, we studied whether PRDX family members interact with HIF-2α in cells. As shown in Figure 1D, PRDX2-V5 and PRDX4-V5 strongly bound to endogenous HIF-2α in hypoxic HeLa cells, whereas PRDX1-V5 and PRDX3-V5 weakly interacted with endogenous HIF-2α. No interaction of HIF-2α with PRDX5-V5 or PRDX6-V5 was detectable in hypoxic HeLa cells. Therefore, in contrast to HIF-1α, HIF-2α mainly binds to PRDX2 and PRDX4 in HeLa cells.

**Figure 2:**
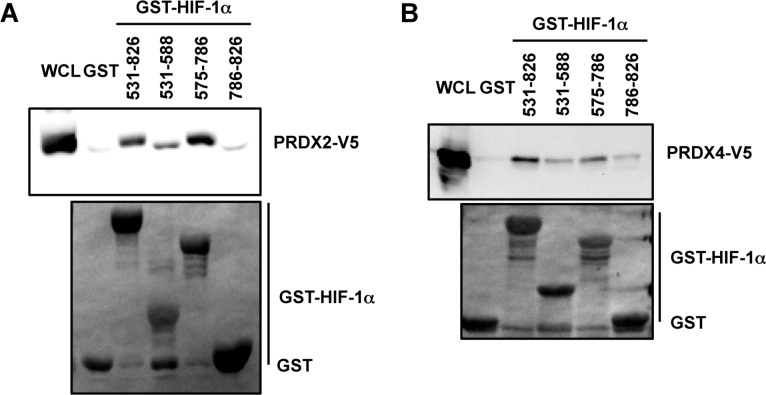
Mapping the PRDX2 and PRDX4 binding domains of HIF-1α **A.** and **B.** HeLa cells were transfected with PRDX2-V5 (A) or PRDX4-V5 (B) vector and WCL was incubated with purified GST or GST-HIF-1α fusion protein in the presence of glutathione-Sepharose beads, followed by immunoblot assays with anti-V5 antibody (upper panels) or Ponceau S staining (lower panels).

Since PRDX2 was identified in our proteomic screen using the transactivation domain of HIF-1α (amino acid residues 531-826) as bait, we further localized the binding of PRDX2 and PRDX4 by glutathione-*S*-transferase (GST) pull-down assays. GST fusion proteins containing HIF-1α amino-acid residues 531-826, 531-588, 575-786, or 786-826 were expressed in bacteria, purified, and incubated with lysates prepared from HeLa-PRDX2-V5 or HeLa-PRDX4-V5 cells in the presence of glutathione-Sepharose beads. As shown in Figure 2A, GST-HIF-1α (531-826) strongly bound to PRDX2-V5, which validated our proteomic screening data. PRDX2-V5 bound to GST-HIF-1α (575-786) with similar avidity, but bound only weakly to GST-HIF-1α (531-588). Compared to GST alone, there was no increased interaction between GST-HIF-1α (786-826) and PRDX2-V5 *in vitro*. These data indicate that PRDX2 binds mainly to HIF-1α (575-786), which encompasses an inhibitory domain of HIF-1α that was previously shown to counteract transactivation domain function [43]. Similarly, PRDX4-V5 showed strongest binding to HIF-1α residues 531-826 and 575-786 (Figure 2B).

### PRDX2 and PRDX4 inhibit HIF transcriptional activity

**Figure 3:**
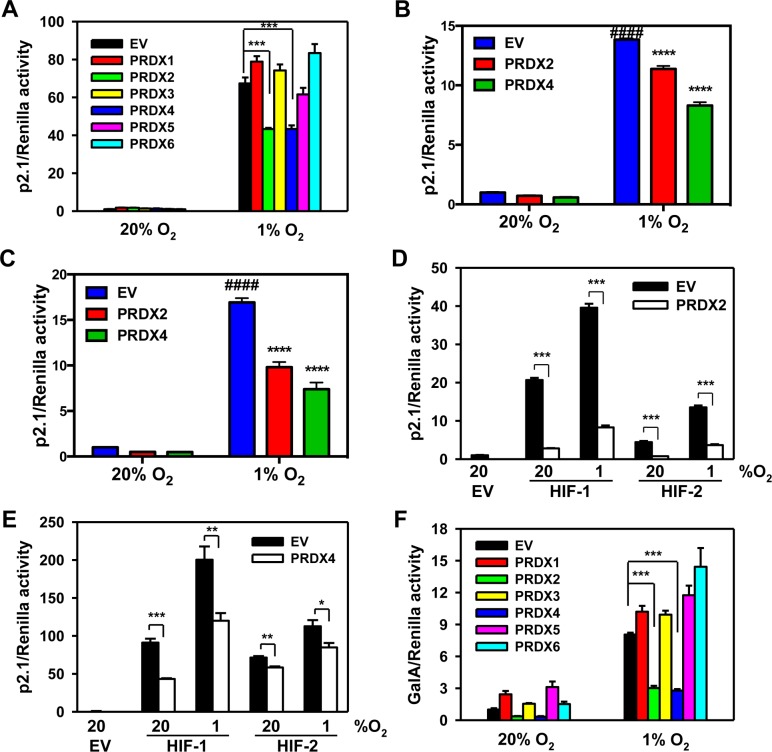
PRDX2 and PRDX4 inhibit the transcriptional activity of HIF-1 and HIF-2 **A.** HeLa cells were transfected with HIF-dependent firefly luciferase (Fluc) reporter p2.1, Renilla luciferase (Rluc) control reporter pSV-Renilla, and empty vector (EV) or PRDX1-6 expression vector, and exposed to 20% or 1% O_2_ for 24 h. Fluc:Rluc activity was normalized to EV at 20% O_2_ (mean ± SEM, *n* = 4). ^***^p < 0.001. **B.** and **C.** Mouse embryo fibroblasts (B) or HEK293T cells (C) were transfected with p2.1, pSV-Renilla, and EV or vector encoding PRDX2-V5 or PRDX4-V5, and exposed to 20% or 1% O_2_ for 24 h. Fluc:Rluc activity was normalized to EV at 20% O_2_ (mean ± SEM, *n* = 3-4). ^####^*p* < 0.0001 *versus* EV at 20% O_2_; ^****^p < 0.0001 *versus* EV at 1% O_2_. **D.** HeLa cells were transfected with p2.1, pSV-Renilla, EV or PRDX2-V5 vector, and HIF-1α or HIF-2α vector, and exposed to 20% or 1% O_2_ for 24 h. Fluc:Rluc activity was normalized to EV at 20% O_2_ (mean ± SEM, *n* = 4). ^***^p < 0.001. **E.** HeLa cells were transfected with p2.1, pSV-Renilla, EV or PRDX4-V5, and HIF-1α or HIF-2α vector and exposed to 20% or 1% O_2_ for 24 h. Fluc:Rluc activity was normalized to EV at 20% O_2_ (mean ± SEM, *n* = 4). ^*^p < 0.05; ^**^p< 0.01; ^***^p < 0.001. **F.** HeLa cells were transfected with pGalA expression vector, Fluc reporter pG5E1bLuc, pSV-Renilla, and EV or PRDX1-6 vector, and exposed to 20% or 1% O_2_ for 24 h. Fluc:Rluc activity was normalized to EV at 20% O_2_ (mean ± SEM, *n* = 4). ^***^p < 0.001.

To determine whether PRDX family members regulate HIF transcriptional activity, HeLa cells were co-transfected with: empty vector (EV) or expression vector encoding a PRDX family member; the HIF-dependent firefly luciferase (Fluc) reporter plasmid p2.1, which contains an HRE from the human *ENO1* gene upstream of SV40 promoter and Fluc coding sequences [8]; and pSV-Renilla, which contains the SV40 promoter alone upstream of Renilla luciferase (Rluc) coding sequences. Transfected cells were exposed to 20% or 1% O_2_ for 24 h. Overexpression of PRDX2-V5 or PRDX4-V5, but not other PRDX family members, significantly decreased HIF transcriptional activity in hypoxic HeLa cells (Figure 3A). Inhibition of HIF transcriptional activity by PRDX2 or PRDX4 was also observed in mouse embryo fibroblasts (Figure 3B) and human embryonic kidney HEK293T cells (Figure 3C). To determine whether PRDX2 and PRDX4 inhibit HIF-1, HIF-2, or both, HeLa cells were co-transfected with p2.1; pSV-Renilla; EV, PRDX2 or PRDX4 expression vector; and expression vector encoding HIF-1α or HIF-2α, and exposed to 20% or 1% O_2_ for 24 h. PRDX2-V5 significantly decreased the transcriptional activity of HIF-1 and HIF-2 (Figure 3D). Similarly, PRDX4-V5 significantly inhibited both HIF-1 and HIF-2 transcriptional activity in co-transfected HeLa cells (Figure 3E).

To further determine whether PRDX2 and PRDX4 have a direct effect on HIF-1α transactivation function, HeLa cells were co-transfected with: pGalA, which encodes the GAL4 DNA-binding domain fused to the HIF-1α transactivation domain (531-826); reporter plasmid pG5E1bLuc, which contains five GAL4-binding sites and a TATA box upstream of Fluc coding sequences [43]; pSV-Renilla; and PRDX expression vector or EV. Transfected cells were exposed to 20% or 1% O_2_ for 24 h. Expression of PRDX2-V5 or PRDX4-V5, but not other PRDX family members, significantly inhibited HIF-1α transactivation domain function in HeLa cells (Figure 3F).

**Figure 4:**
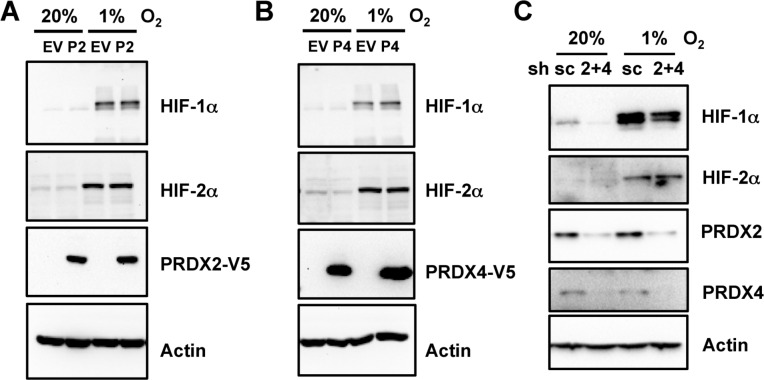
Expression of PRDX2 or PRDX4 does not affect HIF-1α or HIF-2α protein levels **A.** and **B.** HeLa cells were transfected with EV or vector encoding PRDX2-V5 (A, P2) or PRDX4-V5 (B, P4), and exposed to 20% or 1% O_2_ for 24 h. WCL was subject to immunoblot assays with antibody against HIF-1α, HIF-2α, V5, or acin. **C.** HeLa-shSC (sc) and HeLa-shPRDX(2+4) (2+4) cells were exposed to 20% or 1% O_2_ for 24 h in the presence of doxycycline. WCL was subject to immunoblot assays with antibodies against HIF-1α, HIF-2α, PRDX2, PRDX4, and actin.

We next investigated whether PRDX2 or PRDX4 regulates HIF-1α or HIF-2α protein levels. Overexpression of PRDX2-V5 (Figure 4A) or PRDX4-V5 (Figure 4B) did not alter HIF-1α or HIF-2α protein levels in non-hypoxic or hypoxic HeLa cells. Double knockdown of PRDX2 and PRDX4 also had no effect on expression of HIF-1α or HIF-2α levels in HeLa cells (Figure 4C). These data rule out decreased HIF-1α or HIF-2α protein stability as the cause of PRDX2- and PRDX4-mediated inhibition of HIF transcriptional activity.

**Figure 5:**
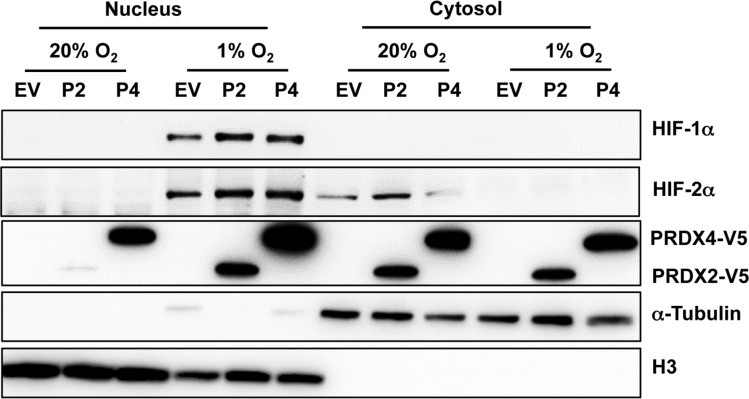
Hypoxia induces the nuclear translocation of PRDX2 and PRDX4 HeLa cells were transfected with vector encoding PRDX2-V5 (P2) or PRDX4-V5 (P4), or empty vector (EV), and exposed to 20% or 1% O_2_ for 48 h. Nuclear and cytosolic fractions were isolated and subject to immunoblot assays with antibodies against HIF-1α, HIF-2α, V5, α-tubulin, and histone H3.

To determine whether PRDX2 or PRDX4 regulates the nuclear localization of HIF-1α and HIF-2α, HeLa cells were transfected with vector encoding PRDX2-V5, PRDX4-V5, or empty vector, exposed to 20% or 1% O_2_ for 48 h, and harvested for preparation of cytosolic and nuclear extracts. Immunoblot assays using antibodies against α-tubulin and histone H3 demonstrated the purity of cytosolic and nuclear fractions, respectively (Figure 5). Analysis of the subcellular fractions revealed that PRDX4-V5 was present in the nucleus and the cytosol, whereas PRDX2-V5 was localized to the cytosol of non-hypoxic HeLa cells (Figure 5). Prolonged hypoxia dramatically increased the nuclear translocation of PRDX2-V5 and PRDX4-V5 in HeLa cells (Figure 5). However, the presence of PRDX2-V5 or PRDX4-V5 did not alter the nuclear translocation of HIF-1α or HIF-2α (Figure 5). These data rule out impaired nuclear translocation of HIF-α subunits as the mechanism by which PRDX2 and PRDX4 interfere with HIF transcriptional activity.

**Figure 6:**
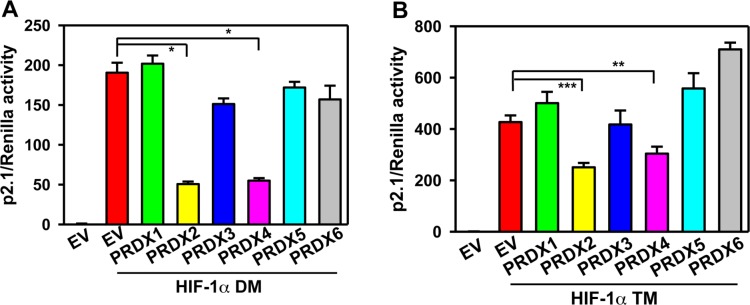
PRDX2 and PRDX4 inhibit HIF-1 independent of hydroxylation **A.** and **B.** HeLa cells were transfected with HIF-dependent Fluc reporter p2.1, control Rluc reporter pSV-Renilla, and EV or vector encoding a PRDX family member and vector encoding HIF-1α(P402A/P564A) (HIF-1α DM) (A) or HIF-1α (P402A/P564A/N803A) (HIF-1α TM) (B). Fluc:Rluc activity was normalized to EV (mean ± SEM, *n* = 4). ^*^p < 0.05; ^**^p < 0.01; ^***^p < 0.001.

To investigate whether PRDX-mediated HIF-1 inhibition depends on proline hydroxylation of HIF-1α, HeLa cells were co-transfected with: p2.1; pSV-Renilla; expression vector encoding PRDX1-6 or EV; and expression vector encoding the double mutant (DM) HIF-1α (P402A/P564A), which is resistant to proline hydroxylation and subsequent VHL-dependent protein degradation [12, 14]. Expression of PRDX2-V5 or PRDX4-V5 significantly reduced HIF-1α-DM-mediated p2.1 luciferase activity (Figure 6A), suggesting that the inhibition of HIF transcriptional activity that is mediated by PRDX2 and PRDX4 is independent of proline hydroxylation.

Next, HeLa cells were co-transfected with: p2.1; pSV-Renilla; expression vector encoding PRDX1-6 or EV; and vector encoding triple mutant (TM) HIF-1α (P402A/P564A/N803A), which is resistant to both proline and asparagine hydroxylation [25, 26]. HIF-1α-TM-mediated p2.1 luciferase activity was significantly inhibited by PRDX2-V5 or PRDX4-V5 (Figure 6B). These data indicate that PRDX2- and PRDX4-mediated HIF-1 inhibition is also independent of asparagine hydroxylation.

### Catalytic activity of PRDX2 and PRDX4 is not required for inhibition of HIF transcriptional activity

**Figure 7:**
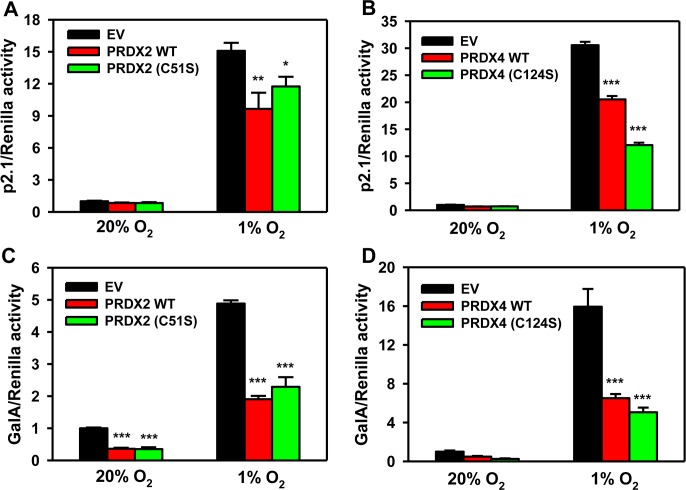
Catalytic activity of PRDX2 and PRDX4 is dispensable for HIF inhibition **A.** HeLa cells were transfected with HIF-dependent Fluc reporter p2.1, control Rluc reporter pSV-Renilla, and EV or vector encoding wild-type (WT) PRDX2-V5 or catalytically inactive PRDX2(C51S)-V5, and exposed to 20% or 1% O_2_ for 24 h. Fluc:Rluc activity was normalized to EV at 20% O_2_ (mean ± SEM, *n* = 4). ^*^p < 0.05,^**^p < 0.01 *versus* EV at 1% O_2_. **B.** HeLa cells were transfected with p2.1, pSV-Renilla, and EV, WT PRDX4-V5, or PRDX4 (C124S)-V5 vector, and exposed to 20% or 1% O_2_ for 24 h. Fluc:Rluc activity was normalized to EV at 20% O_2_ (mean ± SEM, *n* = 4). ^***^p < 0.001 *versus* EV at 1% O_2_. **C.** HeLa cells were transfected with pGalA expression vector, Fluc reporter pG5E1bLuc, Rluc reporter pSV-Renilla, and EV, WT PRDX2-V5 or PRDX2(C51S)-V5 vector, and exposed to 20% or 1% O_2_ for 24 h. Fluc:Rluc activity was normalized to EV at 20% O_2_ (mean ± SEM, *n* = 4). ^***^p < 0.001 *versus* EV at same O_2_ concentration. **D.** HeLa cells were transfected with pGalA vector, pG5E1bLuc, pSV-Renilla, and EV, WT PRDX4-V5, or PRDX4 (C124S)-V5 vector, and exposed to 20% or 1% O_2_ for 24 h. Fluc:Rluc activity was normalized to EV at 20% O_2_ (mean ± SEM, *n* = 4). ^***^p < 0.001 *versus* EV at 1% O_2_.

We next investigated whether the catalytic activity of PRDX2 or PRDX4 is required for HIF suppression. HeLa cells were co-transfected with: p2.1; pSV-Renilla; and expression vector encoding wild-type (WT) PRDX2-V5 or PRDX4-V5, or catalytically inactive PRDX2(C51S)-V5 or PRDX4(C124S)-V5, or EV. PRDX2(C51S)-V5 decreased hypoxia-induced p2.1 luciferase activity, similar to the effect of WT PRDX2-V5 (Figure 7A). The effect of catalytically inactive PRDX4(C124S)-V5 was also similar to WT PRDX4-mediated suppression of HIF activity (Figure 7B). We further confirmed these data by GalA reporter assays, which demonstrated similar effects of WT and catalytically inactive forms of PRDX2 (Figure 7C) and PRDX4 (Figure 7D). These data indicate that the catalytic activity of PRDX2 and PRDX4 is not required for suppression of HIF transcriptional activity.

### PRDX2 and PRDX4 inhibit HIF target gene expression

**Figure 8:**
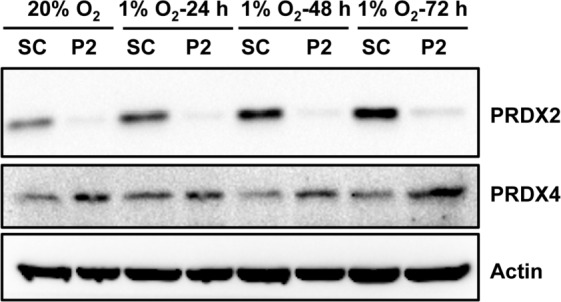
PRDX2 knockdown increases PRDX4 protein levels in HeLa cells HeLa subclones were exposed to 20% or 1% O_2_ for indicated time. Each WCL was subject to immunoblot assays with the indicated antibodies. SC, scrambled control shRNA. P2, PRDX2 shRNA.

**Figure 9:**
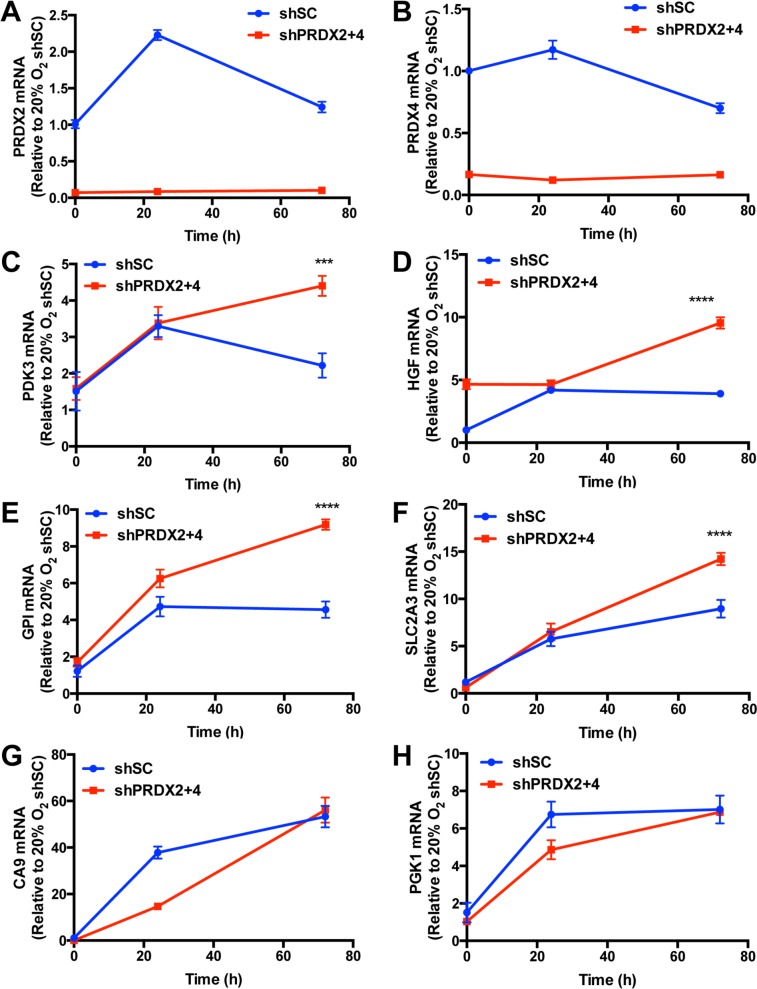
PRDX2 and PRDX4 inhibit the expression of a subset of HIF target genes **A.**-**H.** HeLa-shSC and HeLa-shPRDX2+4 cells were exposed to 20% or 1% O_2_ for 24 or 72 h in the presence of doxycycline. Reverse transcription and quantitative real-time PCR (RT-qPCR) assays were performed using primers specific for PRDX2 (A), PRDX4 (B), PDK3 (C), HGF (D), GPI (E), SLC2A3 (F), CA9 (G), or PGK1 (H) mRNA. The Ct value for each mRNA was normalized to that of 18*S* rRNA and the resulting ratio was further normalized to shSC at 20% O_2_. The fold change of each mRNA is shown as mean ± SEM, *n* = 3. ^***^p < 0.001, ^****^p < 0.0001 *versus* shSC.

We next investigated whether PRDX2 and PRDX4 inhibit the expression of endogenous HIF target genes. We found that PRDX2 knockdown resulted in a compensatory increase in PRDX4 protein levels in non-hypoxic and hypoxic HeLa cells (Figure 8). Considering that increased PRDX4 may counteract the effect of PRDX2 knockdown, we generated doxycycline-inducible PRDX2/PRDX4 double-knockdown cells. HeLa subclones that were stably transfected with vector encoding a scrambled control (SC) short hairpin (sh) RNA (HeLa-shSC) or vectors encoding shRNAs targeting PRDX2 and PRDX4 (HeLa-shPRDX2+4) were exposed to 20% or 1% O_2_ for 24 or 72 h in the presence of doxycycline. The shRNAs efficiently reduced PRDX2 (Figure 9A) and PRDX4 (Figure 9B) mRNA levels. The levels of mRNAs encoded by the HIF target genes *PDK3* (Figure 9C), *HGF* (Figure 9D), *GPI* (Figure 9E), *SLC2A3* (Figure 9F), *CA9* (Figure 9G), and *PGK1* (Figure 9H) were significantly increased after 24 h of hypoxia, but decreased or leveled off after 72 h of continuous hypoxia in HeLa-shSC cells. Double knockdown of PRDX2 and PRDX4 had no effect on the expression of PDK3 (Figure 9C), HGF (Figure 9D), GPI (Figure 9E), or SLC2A3 (Figure 9F) mRNA after 24 h of hypoxia, but significantly increased their mRNA levels after 72 h of hypoxia in HeLa-shPRDX2+4 cells, as compared to HeLa-shSC cells. In contrast, the expression of *CA9* (Figure 9G) and *PGK1* (Figure 9H) was not altered by PRDX2 and PRDX4 double knockdown at 72 h of hypoxia. These data indicate that PRDX2 and PRDX4 selectively inhibit the expression of a subset of HIF target genes in HeLa cells subjected to prolonged hypoxia.

### PRDX2 and PRDX4 block HIF binding to the HREs of a subset of target genes

**Figure 10:**
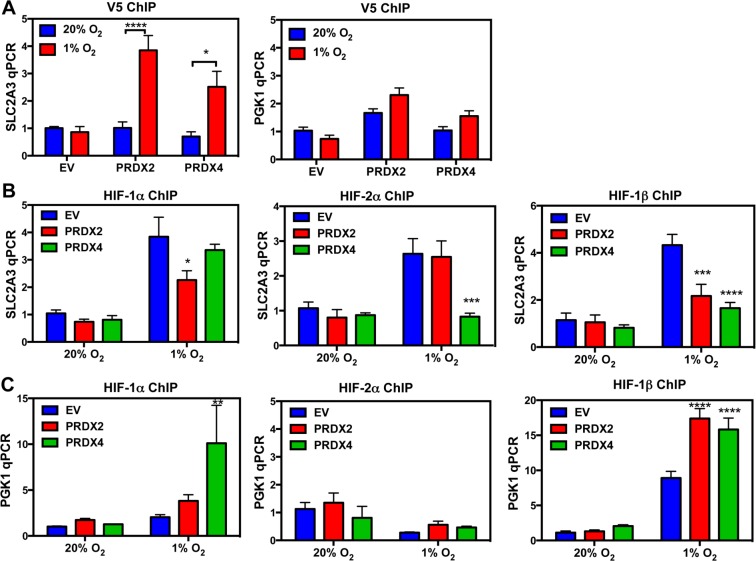
PRDX2 and PRDX4 block HIF-1 and HIF-2 binding to a subset of target genes **A.** HeLa subclones were exposed to 20% or 1% O_2_ for 72 h. Chromatin immunoprecipitation (ChIP) was performed using anti-V5 antibody and DNA was analyzed by qPCR (mean ± SEM, *n* = 3). ^*^p < 0.05; ^****^p < 0.0001. **B.** and **C.** HeLa subclones were exposed to 20% or 1% O_2_ for 72 h. ChIP was performed using anti-HIF-1α, anti-HIF-2α, or anti-HIF-1β antibody and DNA was analyzed by qPCR with primers spanning the HRE of the *SLC2A3* (B) or *PGK1* (C) gene (mean ± SEM, *n* = 3). ^*^p < 0.05, ^**^p < 0.01, ^***^p < 0.001, ^****^p < 0.0001 *versus* EV at 1% O_2_.

To study the molecular mechanisms of PRDX2- and PRDX4-mediated inhibition of HIF-1α transactivation domain function, we first tested whether PRDX2 and PRDX4 are recruited to the HREs of HIF target genes in response to prolonged hypoxia using chromatin immunoprecipitation (ChIP) assays. HeLa subclones that were stably transduced with an empty lentivirus vector (EV) or lentivirus encoding PRDX2-V5 or PRDX4-V5 were exposed to 20% or 1% O_2_ for 72 h. Recruitment of PRDX2-V5 or PRDX4-V5 to the *SLC2A3* gene HRE was significantly increased in HeLa cells exposed to prolonged hypoxia, whereas recruitment to the *PGK1* gene HRE was not significantly increased in hypoxic cells (Figure 10A). Thus, PRDX recruitment to *SLC2A3*, but not to *PGK1* (Figure 10A), was associated with negative regulation of *SLC2A3*, but not *PGK1*, gene expression (Figure 9). We further analyzed whether PRDX2 or PRDX4 affects HIF binding. Overexpression of PRDX2-V5 significantly decreased occupancy of the *SLC2A3* HRE by HIF-1α and HIF-1β, but HIF-2α occupancy of the *SLC2A3* HRE was not affected by PRDX2-V5 overexpression (Figure 10B). PRDX4-V5 overexpression significantly decreased occupancy by HIF-2α and HIF-1β, but not HIF-1α, of the *SLC2A3* HRE (Figure 10B). In contrast, overexpression of PRDX2-V5 or PRDX4-V5 did not inhibit HIF binding to the HRE of the *PGK1* gene (Figure 10C). Taken together, these data indicate that PRDX2 and PRDX4 may act in part by selectively decreasing HIF binding to a subset of target genes, leading to reduced gene transcription under prolonged hypoxia.

### PRDX2 and PRDX4 do not influence RNA polymerase II binding to HREs of HIF target genes

**Figure 11:**
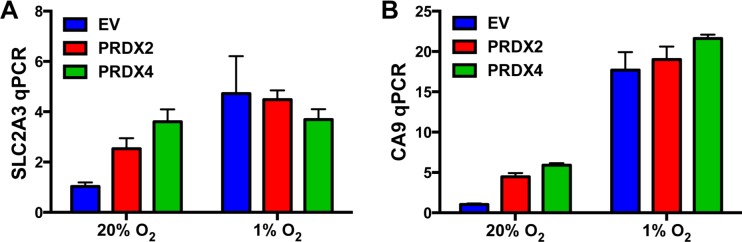
Effect of PRDX2 and PRDX4 on RNA polymerase II phosphorylation and binding to HIF target genes **A.**-**B.** HeLa cells stably transfected with EV or vector encoding PRDX2-V5 or PRDX4-V5 were exposed to 20% or 1% O_2_ for 72 h. Chromatin was immunoprecipitated using anti-RNA polymerase II (pSer5) antibody and DNA was analyzed by qPCR with primers spanning the HRE of the *SLC2A3* (A) or *CA9* (B) gene (mean ± SEM, *n* = 3).

Serine 5 phosphorylation of RNA polymerase II is necessary for gene transcription [44]. To determine whether PRDX2 and PRDX4 regulate the recruitment of phosphorylated RNA polymerase II, we performed ChIP assays using anti-RNA polymerase II (pSer5) antibody in HeLa cells exposed to 20% or 1% O_2_ for 72 h. Hypoxia significantly increased RNA polymerase II (pSer5) binding to the HRE of the *SLC2A3* and *CA9* genes in HeLa cells (Figure 11). Overexpression of PRDX2-V5 or PRDX4-V5 did not influence RNA polymerase II (pSer5) binding (Figure 11). Thus, altered phosphorylation or recruitment of RNA polymerase II does not represent the mechanism by which PRDX2 and PRDX4 inhibit HIF transcriptional activity.

### PRDX2 and PRDX4 do not inhibit the interaction of p300 with HIF-1α

**Figure 12:**
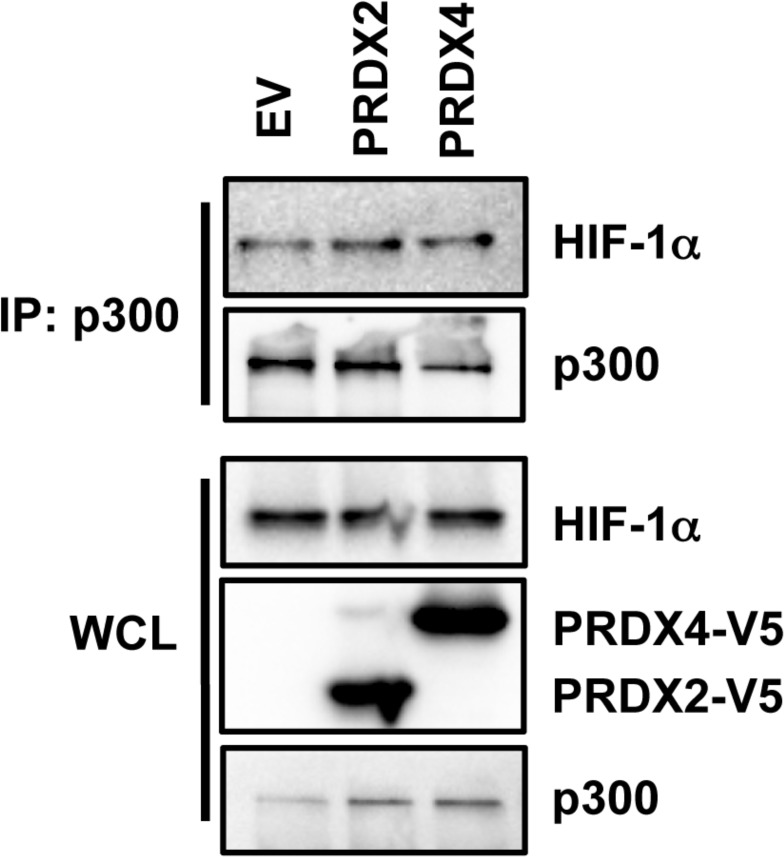
Effect of PRDX2 and PRDX4 on HIF-1α-p300 interaction HeLa cells were transfected with empty vector (EV) or vector encoding PRDX2-V5 or PRDX4-V5, and exposed to 1% O_2_ for 24 h. WCL was subject to IP with anti-p300 antibody, followed by immunoblot assays using antibodies against HIF-1α, V5, and p300.

To determine whether PRDX2 and PRDX4 regulate the recruitment of p300 to HIF-1α, we performed co-IP assays. HeLa cells were transfected with PRDX2-V5 or PRDX4-V5 expression vector, or EV, and exposed to 1% O_2_ for 24 h. As shown in Figure 12, forced expression of PRDX2-V5 or PRDX4-V5 did not alter HIF-1α-p300 interaction in hypoxic HeLa cells. Thus, PRDX2 and PRDX4 do not inhibit the recruitment of p300 to HIF-1α.

### PRDX2 expression is regulated by HIFs

**Figure 13:**
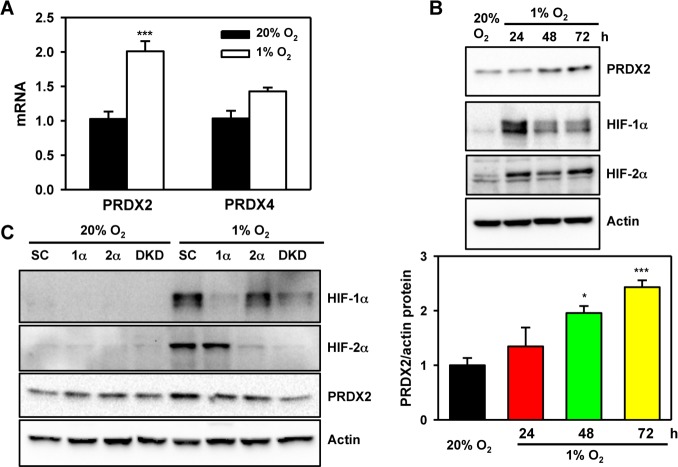
PRDX2 expression is regulated by HIF-1 and HIF-2 **A.** HeLa cells were exposed to 20% or 1% O_2_ for 24 h. RT-qPCR assays were performed using primers specific for the indicated mRNAs. Data are shown as mean ± SEM, *n* = 3. ^***^p < 0.001 *versus* 20% O_2_. **B.** HeLa cells were exposed to 20% or 1% O_2_ for the indicated time. WCLs were subject to immunoblot assays with antibodies against PRDX2, HIF-1α, HIF-2α, and actin. The PRDX2 and actin bands were quantified by densitometry and normalized to 0 h (20% O_2_). Normalized data are shown as mean ± SEM, *n* = 3. ^*^p < 0.05, ^***^p < 0.001 *versus* 20% O2. **C**. HeLa-shSC (SC), HeLa-shHIF-1α(1α), HeLa-shHIF-2α(2α), and HeLa-sh1α+2α (DKD) cells were exposed to 20% or 1% O_2_ for 72 h. WCLs were subject to immunoblot assays with antibodies against PRDX2, HIF-1α, HIF-2α, and actin.

To determine whether HIFs control PRDX expression, we exposed HeLa cells to 20% or 1% O_2_ for 24 or 72 h. Reverse transcription and real-time quantitative PCR (RT-qPCR) assays demonstrated that PRDX2 mRNA levels were significantly increased after 24 h of hypoxia and then decreased to baseline levels at 72 h of hypoxia (Figure 9G and Figure 13A), whereas PRDX4 mRNA levels were not significantly increased after 24 h of hypoxia (Figure 13A). Consistent with mRNA induction, PRDX2 protein expression was induced by hypoxia in a time-dependent manner (Figure 8 and Figure 13B). Knockdown of HIF-1α or HIF-2α alone slightly decreased PRDX2 protein levels in hypoxic HeLa cells, but double knockdown of HIF-1α and HIF-2α prevented hypoxia-induced PRDX2 expression (Figure 13C). These data indicate that both HIF-1 and HIF-2 induce *PRDX2* expression in hypoxic HeLa cells.

**Figure 14:**
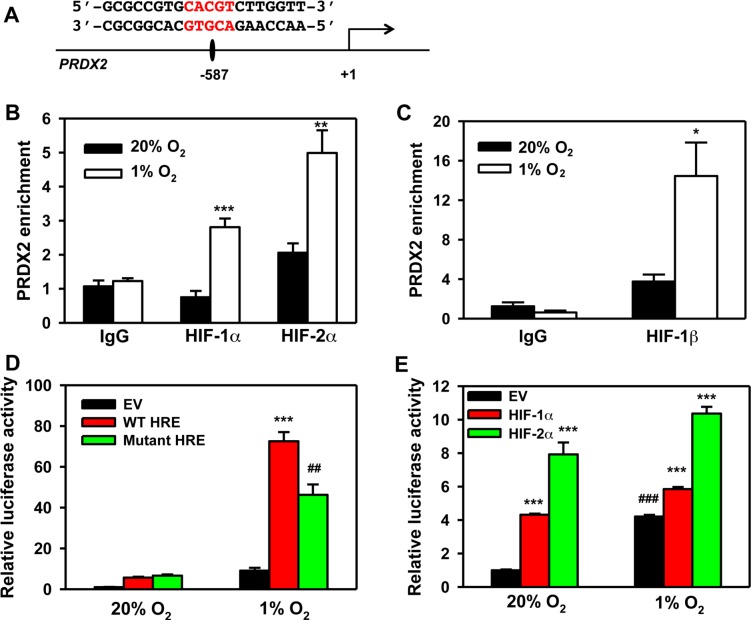
PRDX2 is a direct HIF target gene **A.** Nucleotide sequence of the HIF binding site (5′-ACGTG-3′ shown in red) in the 5′-flanking region of the human *PRDX2* gene (transcription initiation site is designated +1). **B.** and **C.** HeLa cells were exposed to 20% or 1% O_2_ for 24 h. Chromatin was immunoprecipitated using anti-HIF-1α (B), anti-HIF-2α (B), anti-HIF-1β (C) antibody or IgG, and DNA was analyzed by qPCR (mean ± SEM, *n* = 3). ^*^p < 0.05, ^**^p < 0.01, ^***^p < 0.001 *versus* 20% O_2_. **D.** HeLa cells were co-transfected with pGL2 empty vector (EV), pGL2-wild-type *PRDX2* HRE (WT HRE), or pGL2-mutant *PRDX2* HRE (Mut HRE), and pSV-Renilla, and exposed to 20% or 1% O_2_ for 24 h. Fluc:Rluc activity was normalized to EV at 20% O_2_ (mean ± SEM, *n* = 4). ^##^*p* < 0.01 *versus* WT HRE; ^***^p < 0.001 *versus* EV. **E.** HeLa cells were co-transfected with WT HRE, pSV-Renilla, and EV or vector encoding HIF-1α or HIF-2α, and exposed to 20% or 1% O_2_ for 24 h. Fluc:Rluc activity was normalized to EV at 20% O_2_ (mean ± SEM, *n* = 4). ^***^p < 0.001 *versus* EV; ^###^*p* < 0.001 *versus* EV at 20% O_2_.

To determine whether *PRDX2* is a direct HIF target gene, we analyzed the genomic DNA sequence and identified the HIF binding site sequence 5′-ACGTG-3′ on the antisense strand in the 5′-flanking region of the human *PRDX2* gene (Figure 14A). To determine whether HIF binds to this sequence, HeLa cells were exposed to 20% or 1% O_2_ for 24 h and the chromatin was extracted, sheared, and precipitated by antibodies against HIF-1α, HIF-2α, HIF-1β, or IgG. qPCR assays using primers spanning the putative HIF binding site revealed that hypoxia significantly increased occupancy by HIF-1α, HIF-2α, and HIF-1β (Figure 14B-14C). These data indicate that *PRDX2* is a direct HIF-1 and HIF-2 target gene. These results are consistent with the effect of HIF-1α and HIF-2α knockdown on PRDX2 expression (Figure 13C).

Next, we introduced a 54-bp *PRDX2* DNA fragment encompassing the HIF binding site into the pGL2-promoter reporter plasmid upstream of SV40 promoter and Fluc coding sequences and designated the reporter construct pGL-WT-HRE. In HeLa cells co-transfected with pSV-Renilla and pGL-WT-HRE, hypoxia dramatically increased the ratio of Fluc:Rluc activity (Figure 14D). Mutation of the HIF binding site sequence from 5′-ACGTG-3′ to 5′-AAAAG-3′ significantly decreased Fluc:Rluc activity (Figure 14D). Overexpression of HIF-1α or HIF-2α significantly increased pGL2-WT-HRE reporter activity in non-hypoxic and hypoxic HeLa cells (Figure 14E). Taken together, these data indicate that both HIF-1 and HIF-2 bind to an HRE in the 5′ flanking region of the *PRDX2* gene to enhance PRDX2 expression under prolonged hypoxia.

## DISCUSSION

In the present study, we analyzed the role of PRDX family members in regulating HIF transcriptional activity. We found that PRDX2 and PRDX4 interact with HIF-1α and HIF-2α, and inhibit the transcriptional activity of HIF-1 and HIF-2 in multiple cell lines. PRDX2 is localized to the cytosol of non-hypoxic HeLa cells, whereas PRDX4 is present in both the nucleus and the cytosol. Prolonged hypoxia increases the nuclear localization of PRDX2 and PRDX4. As a result, PRDX2 and PRDX4 are recruited to the HREs of a subset of HIF target genes and inhibit their transcription. *PRDX2* transcription is controlled by both HIF-1 and HIF-2 in hypoxic HeLa cells. Thus, prolonged hypoxia regulates PRDX2 at both transcriptional and post-translational levels. Recent studies also demonstrated the nuclear localization of PRDX2, with different subcellular fractions of PRDX2 having distinct functions in regulation of the androgen receptor [45, 46]. Nuclear PRDX2 also inhibits STAT3 transcriptional activity through redox effects [47]. Mutation of the PRDX2 catalytic site abolishes PRDX2 regulation of androgen receptor and STAT3 [47]. In contrast, the enzymatic activity of PRDX2 was not required for inhibition of HIF-1 and HIF-2 transcriptional activity. Our data indicate that the physical interaction of PRDX2 or PRDX4 with the inhibitory domain of HIF-1α is crucial for suppression of HIF-1 transcriptional activity.

We found that PRDX2 and PRDX4 decrease HIF-1 and HIF-2 occupancy of HREs of a subset of HIF target genes. Previous studies from our group and others have suggested that histone modifications, including acetylation and methylation, may regulate HIF binding to HREs [27, 42]. In the current study, we found that p300 recruitment to HIF-1α was not affected by PRDX2 and PRDX4, suggesting that changes in p300-mediated histone acetylation are not involved in PRDX2 and PRDX4-mediated HIF inhibition. Similar results were also found for FHL2-mediated HIF inhibition [29]. RNA polymerase II is required for gene transcription [44]. However, neither PRDX2 nor PRDX4 regulates RNA polymerase II phosphorylation or recruitment to HIF target genes under prolonged hypoxia. Thus, further studies are required to determine the precise mechanism underlying inhibition of HIF transcriptional activity by PRDX2 and PRDX4. Recent studies have reported that HIF-1 stimulates transcriptional elongation [48], suggesting that PRDX2 and PRDX4 may impair elongation by inhibiting HIF binding to a subset of HREs.

There are three types of hypoxia, i.e. acute, intermittent, and prolonged hypoxia, with each type of hypoxia having distinct effects on HIF-1α and HIF-2α [9, 49, 50]. We have previously shown that prolonged hypoxia causes the selective decay of HIF-1α protein, which is mediated at least in part by HSP70- and CHIP-dependent ubiquitination and proteasomal degradation [18]. In the current study, we have demonstrated that PRDX2 and PRDX4 knockdown increases the expression of HIF target genes during prolonged hypoxia. However, only a subset of HIF target genes was controlled by PRDX2 and PRDX4, as previously reported for Reptin [30]. Reduced recruitment of HIFs to the HREs of selected HIF target genes represents a mechanism by which PRDX2 or PRDX4 inhibits HIF-mediated transactivation. Interestingly, PRDX2 and PRDX4 differentially inhibit recruitment of HIF-1α and HIF-2α to the HRE of the *SLC2A3* gene. The mRNA levels of three out of six genes we tested (*SLC2A3*, *GPI*, and *PDK3*) were selectively decreased by PRDX2 and PRDX4 during prolonged hypoxia, suggesting that PRDX2 and PRDX4 may be negative regulators of glucose reprogramming in cancer cells exposed to prolonged hypoxia. Cancer cells exposed to prolonged hypoxia are more aggressive and resistant to radiation and chemotherapy, and differential gene expression is likely to contribute to this phenotype [51]. It will be interesting to determine if PRDX2 and PRDX4 regulate the aggressive phenotype of cancer cells under prolonged hypoxia. As a further measure of complexity, we demonstrate that PRDX2 itself is encoded by a HIF target gene, providing a mechanism for feedback inhibition of genes whose high-level expression is only required during the acute phase of hypoxic exposure.

## MATERIALS AND METHODS

### Plasmid constructs

The complete coding sequences of human PRDX1-6 were amplified from HeLa cell cDNA by PCR and ligated into pcDNA3.1-V5-His (Invitrogen) for transient transfection. PRDX2 or PRDX4 cDNA was also ligated into pLenti4-puro for stable transfection. The catalytically inactive PRDX2(C51S) and PRDX4(C124S) mutants were generated using QuickChange Site-directed Mutagenesis Kit (Stratagene). PRDX2 and PRDX4 shRNA oligonucleotides were ligated into Teton-pLKO (Addgene #21915). HIF-1α and HIF-2α shRNAs were cloned into pLKO.1. The oligonucleotide sequences of shRNAs are shown in Table 1. Other constructs have been described previously [41, 42]. The DNA sequences of all recombinant plasmids were confirmed by nucleotide sequence analysis.

**Table 1:** Oligonucleotide sequence for shRNAs, qRT-PCR, and ChIP

**Oligonucleotide sequence of shRNAs**	
shPRDX2	5′ CCTTCGCCAGATCACTGTTAA 3′
shPRDX4	5′ CCACACTCTTAGAGGTCTCTT 3′
shHIF-1α	5′ TGCTCTTTGTGGTTGGATCTA 3′
shHIF-2α	5′ GGAGACGGAGGTGTTCTATT 3′
**Primers used in qRT-PCR assays**	
PRDX2	Fw:5′ GAAGCTGTCGGACTACAAAGG 3′ Rev: 5′ TCGGTGGGGCACACAAAAG 3′
PRDX4	Fw: 5′ AGAGGAGTGCCACTTCTACG 3′ Rev: 5′ GGAAATCTTCGCTTTGCTTAGGT 3′
HGF	Fw: 5′ GCTATCGGGGTAAAGACCTACA 3′ Rev: 5′ CGTAGCGTACCTCTGGATTGC 3′
GPI	Fw: 5′ CCGCGTCTGGTATGTCTCC 3′ Rev: 5′ CCTGGGTAGTAAAGGTCTTGGA 3′
SLC2A3	Fw: 5′ GCTGGGCATCGTTGTTGGA 3′ Rev: 5′ GCACTTTGTAGGATAGCAGGAAG 3′
PDK3	Fw: 5′ CGCTCTCCATCAAACAATTCCT 3′ Rev: 5′ CCACTGAAGGGCGGTTAAGTA 3′
CA9	Fw: 5′ GGATCTACCTACTGTTGAGGCT 3′ Rev: 5′ CATAGCGCCAATGACTCTGGT 3′
PGK1	Fw: 5′ TGGACGTTAAAGGGAAGCGG 3′ Rev: 5′ GCTCATAAGGACTACCGACTTGG 3′
18S rRNA	Fw: 5′ CGGCGACGACCCATTCGAAC 3′ Rev: 5′ GAATCGAACCCTGATTCCCCGTC 3′
**Primers used in ChIP assays**	
SLC2A3 HRE	Fw: 5′ TCAAGTCTTCAAGAAAGATCTAGG 3′ Rev: 5′ GACCCAGAGATGCTGTAATG 3′
PGK1 HRE	Fw: 5′ TCTCGCACATTCTTCACGTCCGTT 3′ Rev: 5′ TAGTGAGACGTGCGGCTTCCGTTT 3′
PRDX2 HRE	Fw: 5′ CGTGCACGTCTTGGTTC 3′ Rev: 5′ CTAGACGCACGGACGAT 3′

### Cell culture and transfection

HeLa cells, mouse embryo fibroblasts, and HEK293T cells were cultured in DMEM supplemented with 10% heat-inactivated fetal bovine serum at 37°C in a 5% CO_2_/95% air incubator. Cells were transfected using PolyJet DNA, according to the manufacturer's protocol (SignaGen). HeLa cells stably transfected with Teton-shPRDX2 and Teton-shPRDX4 vectors were treated with doxycycline (0.5 μg/ml) and sodium pyruvate (10 mM). All cells were verified as *Mycoplasma* free by PCR.

### Hypoxia

Cells were placed in a modular incubator chamber (Billups-Rothenberg) flushed with a gas mixture containing 1% O_2_, 5% CO_2_, and balance N_2_ and incubated at 37°C.

### Lentivirus production

The lentiviruses encoding shRNA (shSC, shHIF-1α, shHIF-2α, shPRDX2, or shPRDX4), PRDX2, or PRDX4 were generated by transfection of HEK293T cells with transducing vector and packaging vectors pMD2.G and psPAX2. After 48 h, virus particles in the medium were harvested, filtered, and transduced into HeLa cells.

### GST pull-down assays

GST and GST-HIF-1α fusion proteins were expressed in *E. coli* BL21-Gold (DE3) and purified [41]. Equal amounts of GST and GST-HIF-1α fusion proteins immobilized on glutathione-Sepharose beads were incubated overnight with whole cell lysates. After washing three times, the bound proteins were fractionated by SDS-PAGE, followed by immunoblot assays.

### IP and immunoblot assays

Whole cell lysates were prepared in modified RIPA buffer [50 mM Tris-HCl (pH 7.5), 1 mM β-mercaptoethanol, 150 mM NaCl, 1% Igepal, and protease inhibitor cocktail] and incubated overnight with the following antibodies (catalog number and supplier): HIF-1α (sc-10790, Santa Cruz); V5 epitope (NB600-381, Novus Biologicals), HIF-2α (NB100-122, Novus Biologicals), or p300 (NB500-161, Novus Biologicals) in the presence of protein A Sepharose beads (NBP1-97240, Novus Biologicals). After washing three times, the bound proteins were fractionated by SDS-PAGE, followed by immunoblot assays using antibodies against the following: HIF-1α (610958, BD Bioscience); HIF-2α (NB100-122, Novus Biologicals); and V5 epitope (R960-25, Invitrogen). Other antibodies used in immunoblot assays recognized PRDX2 (H00007001-M01, Novus Biologicals) and PRDX4 (NBP2-19778, Novus Biologicals), and GST (sc-459, Santa Cruz) and actin (sc-1616, Santa Cruz).

### Subcellular fractionation assays

HeLa cells were lysed in hypotonic buffer [10 mM HEPES/KOH (pH 7.5), 10 mM KCl, 1.5 mM MgCl_2_, 1 mM K_2_EDTA, 1 mM EGTA, 0.1% Igepal, 1 mM DTT, and protease inhibitor cocktail] in a Dounce homogenizer (30 strokes). Intact cells were removed by centrifugation at 50 *g* for 10 min. The nuclei were collected by centrifugation at 800 *g* for 10 min, washed, and lysed in isotonic buffer (hypotonic buffer plus 250 mM sucrose) by sonication to prepare the nuclear fraction. The supernatant was centrifuged at 13,000 *g* for 10 min and the resulting supernatant was taken as the cytosolic fraction [41].

### RT-qPCR assays

Total RNA was isolated using Trizol (Invitrogen) and treated with DNase I (Ambion). RT-qPCR assays were performed as described [41]. Primer sequences are shown in Table 1.

### Luciferase reporter assays

HeLa cells, mouse embryo fibroblasts, or HEK293T cells were seeded onto 48-well plates and transfected with p2.1 [8], PRDX2 HRE reporter, or pGalA and pG5E1bLuc [43]; pSV-Renilla; and pcDNA3.1-V5, pcDNA3.1-PRDX1-V5, pcDNA3.1-PRDX2-V5, pcDNA3.1-PRDX2(C51S)-V5, pcDNA3.1-PRDX3-V5, pcDNA3.1-PRDX4-V5, pcDNA3.1-PRDX4(C124S)-V5, pcDNA3.1-PRDX5-V5, pcDNA3.1-PRDX6-V5, pCMV-FLAG-HIF-1α, pCMV-FLAG-HIF-1α (P402A/P564A), pCMV-FLAG-HIF-1α (P402A/P564A/N803A), or pcDNA-HIF-2α. Three or four independent transfections were performed. Cells were exposed to 20% or 1% O_2_ for 24 h. Fluc and Rluc activities were determined using the Dual-Luciferase Assay System (Promega) and the Fluc:Rluc ratio was determined.

### ChIP assays

Cells were exposed to 20% or 1% O_2_ for 24 or 72 h, cross-linked with 1% formaldehyde for 20 min at 37°C, and quenched in 0.125 M glycine. DNA immunoprecipitated from the sonicated cell lysates was quantified by SYBR Green Real-time PCR (Bio-Rad) [41]. The following antibodies were used: V5 (66007-1-lg, Proteintech), HIF-1α (sc-10790, Santa Cruz), HIF-1β (NB100-124, Novus Biologicals), HIF-2α (NB100-122, Novus Biologicals), and RNA polymerase II (pSer5) (NB200-598, Novus Biologicals). PCR primer sequences are shown in Table 1.

### Statistical analysis

Data are expressed as mean ± SEM. Differences were analyzed by Student's *t*-test between two groups, or one-way or two-way analysis of variance between multiple groups. *P* < 0.05 was considered significant.
